# Supplementary material for: Epidemiological description of 529 families referred for French transcultural psychotherapy: A decade of experience
Source: PLoS One. 2020 Aug 4;15(8):e0236990. doi: 10.1371/journal.pone.0236990 (PMC7402487; doi:10.1371/journal.pone.0236990)
Supplement: S1 Table — (DOCX) [file pone.0236990.s001.docx]

**S1 Table – Characteristics of the entire follow-up population**

|  | **Total population**  **% (N)** | **Sub-Saharan Africa**  **% (N)** | **Asia**  **% (N)** | **MENA**  **% (N)** | **Europe**  **% (N)** | **Caribbean**  **% (N)** |
| --- | --- | --- | --- | --- | --- | --- |
| **Age** |  |  |  |  |  |  |
| Children | **70.8 (213)** | 69.4 (120) | 81.7 (49) | 74.4 (29) | 53.3 (8) | 50 (7) |
| *Including UM* | ***6.6 (14)*** | *5 (6)* | *14.3 (7)* | *0* | *0* | *14.3 (1)* |
| Adults | **29.2 (88)** | 30.6 (53) | 18.3 (11) | 25.6 (10) | 46.7 (7) | 50 (7) |
| **Children gender** |  |  |  |  |  |  |
| Boys | **52 (111)** | 51.7 (62) | 51 (25) | 55.2 (16) | 50 (4) | 57.1 (4) |
| Girls | **48 (102)** | 48.3 (58) | 49 (24) | 44.8 (13) | 50 (4) | 42.9 (3) |
| **Adults gender** |  |  |  |  |  |  |
| Male | **19.3 (17)** | 22.6 (12) | 9.1 (1) | 20 (2) | 28.6 (2) | 0 |
| Female | **80.7 (71)** | 77.4 (41) | 90.9 (10) | 80 (8) | 71.4 (5) | 100 (7) |
| **Cultural problem** |  |  |  |  |  |  |
| Traditional theory | **34.9 (105)** | 43.4 (75) | 11.7 (7) | 25.6 (10) | 6.7 (1) | 85.7 (12) |
| Family conflicts | **23.3 (70)** | 20.2 (35) | 26.7 (16) | 25.6 (10) | 46.7 (7) | 14.3 (2) |
| Cultural misunderstandings | **18.3 (55)** | 14.5 (25) | 35 (21) | 10.3 (4) | 33.3 (5) | 0 |
| Traumatic migration | **16.9 (51)** | 16.8 (29) | 21.7 (13) | 18 (7) | 13.3 (2) | 0 |
| Traditional care | **6.6 (20)** | 5.2 (9) | 5 (3) | 20.5 (8) | 0 | 0 |
| **Presence of an interpreter** |  |  |  |  |  |  |
| Yes | **53.5 (161)** | 42.2 (73) | 88.3 (53) | 56.4 (22) | 46.7 (7) | 42.9 (6) |
| No | **46.5 (140)** | 57.8 (100) | 11.7 (7) | 43.6 (17) | 53.3 (8) | 57.1 (8) |
| **Main psychiatric symptom** |  |  |  |  |  |  |
| Depressive symptoms | **37.5 (113)** | 36.4 (63) | 38.3 (23) | 41 (16) | 33.3 (5) | 42.9 (6) |
| Psychotic symptoms | **21.9 (66)** | 26 (45) | 15 (9) | 18 (7) | 6.7 (1) | 28.6 (4) |
| Externalized symptoms | **19.3 (58)** | 19.1 (33) | 16.7 (10) | 20.5 (8) | 40 (6) | 7.1 (1) |
| Developmental symptoms | **11 (33)** | 9.3 (16) | 18.3 (11) | 7.7 (3) | 20 (3) | 0 |
| Anxious or traumatic symptoms | **10.3 (31)** | 9.3 (16) | 11.7 (7) | 12.8 (5) | 0 | 21.4 (3) |
| **Total** | **301** | **173** | **60** | **39** | **15** | **14** |

*Legend: UM: unaccompanied minor; %: percentage; N: population.*
