# Supplementary material for: Epidemiological description of 529 families referred for French transcultural psychotherapy: A decade of experience
Source: PLoS One. 2020 Aug 4;15(8):e0236990. doi: 10.1371/journal.pone.0236990 (PMC7402487; doi:10.1371/journal.pone.0236990)
Supplement: Attachment — Submitted filename: answer20200322.docx [file pone.0236990.s002.docx]

**Response to Reviewers**

PONE-D-19-34239
An epidemiological description of 529 families treated by the French method of Transcultural Psychotherapy: A decade of experience

**Reviewer #1**

***The study is well-done, scientifically sound and addresses an important topic for which the authors should be congratulated. However, I strongly recommend authors to enrich the article with more scholarly references.
See my suggestions below:***

We thank reviewer #1 for these remarks. Please find below the response to each point raised.

Q1. INTRODUCTION Authors should provide the readers with a broader background and state-of-art of the topic under study: for example, when they speak of mental and psychological issues affecting migrants, they could discuss the following articles Somatic perception, cultural differences and immigration: results from administration of the Modified Somatic Perception Questionnaire (MSPQ) to a sample of immigrants. Bragazzi NL, Puente GD, Natta WM. Psychol Res Behav Manag. 2014 Jun 12;7:161-6; Effects of acculturation, coping strategies, locus of control, and self-efficacy on chronic pain: study of Chinese immigrant women in Italy - insights from a thematic field analysis. Re TS, Bragazzi NL, Siri A, Cisneros Puebla C, Friese S, Simões M, Candau J, Khabbache H. J Pain Res. 2017 Jun 6;10:1383-1390; Front Psychol. 2019 Jan 17;9:2792. doi: 10.3389/fpsyg.2018.02792. eCollection 2018. A Clinical-Psychological Perspective on Somatization Among Immigrants: A Systematic Review. Lanzara R, Scipioni M, Conti C.

R1. The background has been expanded thanks to the references proposed by the reviewer.

Modifications in the text:

Introduction

Migrants express distress in many different ways [11], show a greater tendency to somatization [12], present higher risks of chronic pain [13], and are at higher risk of misdiagnosis than nonmigrants [14].

References

Choi H. Understanding Adolescent Depression in Ethnocultural Context: Adv Nurs Sci. 2002;25: 71–85. doi:10.1097/00012272-200212000-00006

Bragazzi N, Del Puente G, Natta WM. Somatic perception, cultural differences and immigration: results from administration of the Modified Somatic Perception Questionnaire (MSPQ) to a sample of immigrants. Psychol Res Behav Manag. 2014; 161. doi:10.2147/PRBM.S55393

Re T, Bragazzi N, Siri A, Cisneros Puebla C, Friese S, Simões M, et al. Effects of acculturation, coping strategies, locus of control, and self-efficacy on chronic pain: study of Chinese immigrant women in Italy &ndash; insights from a thematic field analysis. J Pain Res. 2017;Volume 10: 1383–1390. doi:10.2147/JPR.S115449

Baker FM. Diagnosing depression in African Americans. Community Ment Health J. 2001;37: 31–38.

Q2. DISCUSSION Authors could enrich the discussion comparing their results with the literature and quoting relevant articles, especially systematic reviews such as: Front Psychol. 2019 Jan 17;9:2792. doi: 10.3389/fpsyg.2018.02792. eCollection 2018. A Clinical-Psychological Perspective on Somatization Among Immigrants: A Systematic Review. Lanzara R, Scipioni M, Conti C.

R2. We have revised the discussion in light with these elements from the literature pointed out by the reviewer.

Modifications in the text:

Discussion

This result is in line with the literature: for example, Lanzara et al. found conflicting results about differences in prevalence of somatization between cultural groups [46].

…

Betancourt et al. found a similar retention rate of 82% in a randomized trial testing a family-based mental health promotion among Somali Bantu and Bhutanese refugees [54]. But the comparison is subject to caution because of the difference in the design of the study (interventional study vs observational study).

References

Lanzara R, Scipioni M, Conti C. A Clinical-Psychological Perspective on Somatization Among Immigrants: A Systematic Review. Front Psychol. 2019;9. doi:10.3389/fpsyg.2018.02792

Betancourt TS, Berent JM, Freeman J, Frounfelker RL, Brennan RT, Abdi S, et al. Family-Based Mental Health Promotion for Somali Bantu and Bhutanese Refugees: Feasibility and Acceptability Trial. J Adolesc Health. 2020;66: 336–344. doi:10.1016/j.jadohealth.2019.08.023

Buhmann CB, Carlsson J, Mortensen EL. Satisfaction of trauma-affected refugees treated with antidepressants and Cognitive Behavioural Therapy. Torture J. 2018;28: 118–129. doi:10.7146/torture.v28i2.106879

**Reviewer #2**

Q1. I have reviewed the manuscript “An epidemiological description of 529 families treated by the French method of Transcultural Psychotherapy: A decade of experience”. The study aims to describe the patients referred for Transcultural Psychotherapy (TPT) in Paris and its suburbs over the past decade and to assess TPT in terms of patient adhesion, attendance, and duration of care. However, the overall purpose of this paper and its broader relevance remain unclear. This together with several other shortcomings further described below and most importantly the lack of correlation between results, discussion and conclusion, leads me to recommend rejection of this paper.

A1. We apologize for the lack of clarity in the purpose of our manuscript. The overall purpose was to describe both the patients referred to TPT and their care pathway in the specific setting of TPT. We hope this revised version of our manuscript will meet the reviewer’s expectations.

Q2. Introduction: The paper concerns TPT, a therapy that the authors state is widely used in French speaking countries. The references cited (ref. 16-19) are however all written by the last author of the present study and are all except one in French which limit the accessibility to a non-French speaking audience. In the present paper, there a no description of the content of TPT or reference to manuals. This makes it difficult to compare the result of the present study to others and limits the clinical usefulness.

A2. TPT is a type of psychotherapy developed by Moro et al and is increasingly widely used in France. As it is not a common or standard care, it must nonetheless be considered experimental. Two manuals are available in French (*Moro MR, Noë Q de L, Mouchenik Y. Manuel de psychiatrie transculturelle : Travail clinique, travail social. Paris: Pensée sauvage; 2006. 421 p. ; 1. Moro MR. Guide de psychothérapie transculturelle : Soigner les enfants. Paris: In Press; 2020. (Old’up Invente)*) and Italian (Moro MR, De la Noë Q, Mouchenik Y, Baubet T. Eds Manuale di psichiatria transculturale. Dalla clinica alla società. Milano, Franco Angeli, 2009 (Italia)), but no English version has yet been published. Nonetheless, several scientific papers in English describe TPT (see, for example, *Sturm G, Baubet T, Moro MR. Culture, trauma, and subjectivity: The French ethnopsychoanalytic approach. Traumatology. 2010;16(4):27‑38*; *Sturm G, Guerraoui Z, Bonnet S, Gouzvinski F, Raynaud J-P. Adapting services to the needs of children and families with complex migration experiences: The Toulouse University Hospital’s intercultural consultation. Transcultural Psychiatry. 2017;54(4):445‑65; Sturm G, Nadig M, Moro MR. Current developments in French ethnopsychoanalysis. Transcultural Psychiatry. 2011;48(3):205‑27*). These references have been added to the manuscript

Modifications in the text:

References

Moro MR. Transcultural psychotherapy of migrants' children [Psychothérapie transculturelle des enfants de migrants]. Dunod; 1998.

Sturm G, Heidenreich F, Moro MR. Transcultural Clinical Work with Immigrants, Asylum Seekers and Refugees at Avicenne Hospital, France. Int J Migr Health Soc Care. 2009;4: 33–40. doi:10.1108/17479894200800022

Sturm G, Baubet T, Moro MR. Culture, trauma, and subjectivity: The French ethnopsychoanalytic approach. Traumatology. 2010;16: 27–38. doi:10.1177/1534765610393183

Moro MR. Guide de psychothérapie transculturelle : Soigner les enfants. Paris: In Press; 2020.

Sturm G, Guerraoui Z, Bonnet S, Gouzvinski F, Raynaud J-P. Adapting services to the needs of children and families with complex migration experiences: The Toulouse University Hospital’s intercultural consultation. Transcult Psychiatry. 2017;54: 445–465. doi:10.1177/1363461517724277

Sturm G, Nadig M, Moro MR. Current developments in French ethnopsychoanalysis. Transcult Psychiatry. 2011;48: 205–227. doi:10.1177/1363461511402868

Q3. Materials and Methods: The authors divide their population into three categories: “Referral rejected”, “initiated treatment” and “effective treatment”. However, the only thing that separates the two latter groups are the number of sessions attended. I find it highly problematic that the authors mix up treatment adherence and effect, a confusion that remains throughout the paper. Therefore, would strongly advice against using the word “effective”, as there are no measures of treatment effect in this paper.

A3. We thank the reviewer for this important remark. As said, our results address only attendance but say nothing about the effectiveness of the treatment (except the fact that attendance at therapy sessions is a prerequisite for therapeutic efficacy). We agree that the term ‘effective’ could lead to confusion and modified it. Depending on the context, we have replaced effective by adherent, actually underway, - treatment that is underway, engaged, in gear, moving forward, operational, — a collaboration, therapy that is operative, functioning, working,

Modifications in the text:

Please refer to the entire manuscript

Q4. Results: The result section is lengthy, and it is again difficult to clearly see the relevance of part of the provided information. For example, on p. 9, the author provides a very thorough description of the origin of different groups of children, only to conclude that cultural area of origin was not associated with age or gender.

A4. One of the purposes of the manuscript was to describe in detail the patients received in TPT. We think that the description of the origins of the children is an important part of our results, as it shows the variety of cultural contexts addressed by TPT – unlike other settings such as *ethnospecific clinics (*See for example Ton H, Koike A, Hales RE, Johnson JA, Hilty D. A Qualitative Needs Assessment for Development of a Cultural Consultation Service. Transcultural Psychiatry. sept 2005;42(3):491‑504). Similarly, the absence of association between origin and age or gender highlights the diversity of the situations referred for this form of therapy. We have reorganized the results section to simplify it without sacrificing what we think it important information.

Modifications in the text:

Please see the revised results section.

Q5. Main psychiatric symptoms are mentioned on. p. 11, but it is not anywhere stated how information about these were obtained (for example whether if they origin from self-ratings or clinical interviews) which is of major importance for the reliability of these data.

A5. We apologize for this omission. The method of data collection for psychiatric symptoms is now specified in the methods section.

Modifications in the text:

Materials and Methods

The psychiatric symptoms described here are those explicitly reported by the first line professional in the referral request.

Q6. Table 4 (p. 12) solely concerns the “effective” treatment group, again with no explanation of the rationale behind this presentation of findings.

A6. We have renamed the ‘effective treatment group’ the "engaged treatment group" and think this clarification makes this point more understandable. We identified/defined attendance at 3 sessions as an indicator of patient investment in the therapy. Most of the time, the first two TPT sessions are devoted to the therapist group getting to know the patient and family, and vice versa, and developing a therapeutic alliance that will encourage adherence and enable effective treatment. We have provided these details in the methods section.

Modifications in the text:

Materials and Methods

The decision of the three-session cut-off was made by a group of clinicians specialized in TPT on the basis of clinical arguments: the two first TPT sessions are devoted to the therapist group getting to know the patient and family, and vice versa, and to developing a therapeutic alliance that will encourage adherence and engagement and will enable effective treatment. From the third session on, these experts consider that therapeutic work has been engaged, is underway.

Q7. Discussion: The third and fourth section of the discussion (P. 13-14) are comparing first and second generation of migrant children and has seemingly little to do with the results of the present study.

A7. The aim of these two parts was to explain the overrepresentation of children in our sample. We think it is important to separate explanations for first- and second-generation migrants, for their psychopathology is not the same. We apologize for the unclearness of these sections and have revised them to make these points more explicit.

Modifications in the text:

Please see the revised discussion section

Q8. From p. 15 and onwards the author starts discussion their own results – but the argumentation in often unclear. For example, on p. 15, bottom section the author states: “it appears clear that rate among the patients with effective treatment is lower than in any other psychotherapeutic setting”. However, the “effective” treatment group are defined by persons who have continued treatment past 2nd session and hence it makes no sense to say that the early dropout in this group was low.

R8. We apologize for this error; we meant the "initiated" group (which covers very diverse situations: dropouts, but also inappropriate referrals, and quite often, issues that could be resolved quickly). Even if we consider all of the cases included in this group to be early dropouts, they account for 30% of the total population — which is a relatively low dropout rate.

Modifications in the text:

Discussion

Nevertheless, it appears clear that the early dropout rate among the patients ~~with effective treatment (≥ 3 sessions)~~ is lower than in any other psychotherapeutic setting, despite the additional risk factors among migrant patients

Q9. The authors furthermore explain the higher rates of children in TPT with the sentence “the experience of migration stresses them more strongly than their parents” (p. 13), a statement that is not at all accounted for by neither the data (where 75% of the children were second generation migrants) or by the general literature. The overrepresentation of children in the present study would likely be due to other factors such as referral/rejection patterns (though little information is given on the rejected patients, it could be the case that the majority of those referred to individual therapy would be adults) and/or the content of the intervention.

R9. As the reviewer knows, the experience of migration isn’t strictly limited to the travel: how they are received and the complexity of the acculturation process weaken migrant parents and affect their children, even those born in the host country. We provided data supporting the affirmation that “the experience of migration stresses them more strongly than their parents” throughout this section (see, for example, Belhadj Kouider E, Koglin U, Petermann F. Emotional and behavioral problems in migrant children and adolescents in Europe: a systematic review. Eur Child Adolesc Psychiatry. 2014;23: 373–391. doi:10.1007/s00787-013-0485-8). We have nonetheless modified the sentence to qualify our statement and expanded the limitations of the study in this regard.

Modifications in the text:

Discussion

These points may explain both the higher TPT referral rates for minors and the sex-ratio differential among adults. The latter result may also be explained by the more general underutilization of mental health services by men – native born or immigrant, in France and in many other countries.

Strengths & limits of the study

Furthermore, the lack of data about rejected patients limits the possibilities of understanding the higher rates of children treated by TPT.

Q10. As for attendance the authors state that it is hard to find comparable studies that provide attentions rates. However, there are studies to be found with comparable populations and attendance rates (Betancourt et al., 2019). The discussion of the reasons for the high attendance rate seems quite speculative and does not mention the possibility that attention rate is (relatively) high because the therapy is brief and not very intensive (meetings every 7th week!).

R10. We have added comparisons with the studies proposed by the reviewer. However, we disagree with the statement that "therapy is brief and not very intensive". For patients whose treatment is considered engaged (at least three sessions), the median duration of the follow-up is 18 months, with nearly 11 one-hour sessions of therapy.

Modifications in the text:

Discussion

Betancourt et al. found a similar retention rate of 82% in a randomized trial testing a family-based mental health promotion among Somali Bantu and Bhutanese refugees [54]. But the comparison is subject to caution because of the difference in the design of the study (interventional study vs observational study).

References

Betancourt TS, Berent JM, Freeman J, Frounfelker RL, Brennan RT, Abdi S, et al. Family-Based Mental Health Promotion for Somali Bantu and Bhutanese Refugees: Feasibility and Acceptability Trial. J Adolesc Health. 2020;66: 336–344. doi:10.1016/j.jadohealth.2019.08.023

Buhmann CB, Carlsson J, Mortensen EL. Satisfaction of trauma-affected refugees treated with antidepressants and Cognitive Behavioural Therapy. Torture J. 2018;28: 118–129. doi:10.7146/torture.v28i2.106879

Q11. The limitation section is short, and the authors seem unaware of the major limitations of their study.

R11. The description of the strengths & limitations of the study has been expanded in accordance with the remarks of all three reviewers.

Modifications in the text:

Please see the new strengths and limitations subsection of the discussion.

Q12. Conclusion: None of the conclusions drawn seem to be supported by the data. Again, it is stated that the overrepresentation of children is due to their greater psychological vulnerability and greater vigilance about the suffering of children, conclusions that cannot be drawn based on available data (please see above). Even more problematic is the link the authors draw between attendance and effect. The author states (p. 17): “The high level of attention, adherence, and engagement in treatment indicates that TPT is an effective method addressing the complex symptoms experienced by migrant families.” This conclusion is downright wrong since no measures of treatment effect are applied in the present study. While adherence may to some extent be correlated to treatment satisfaction, it has nothing to do with effect and patients can easily be satisfied and adherent with their treatment, despite having limited outcomes (Buhmann et al., 2018). Based on the above, I find that the paper does not fulfill PLOS One publication criteria no. 4 (Conclusions are presented in an appropriate fashion and are supported by the data) and furthermore lacks the quality and relevance to a broader audience expected from a PLOS ONE publication, which is why I recommend rejection.

R12. Please see the responses above to each specific point. We have modified the conclusion to qualify the sentences, in particular making it clear when we are presenting hypotheses.

Modifications in the text:

Strengths & limits of the study

Finally, and most important, the data we could measure — attendance, duration of treatment, or number of sessions — say nothing about the content of the therapy or its effectiveness [59]. Hypotheses about an association between attendance, adherence, and success of the therapy need to be examined in relation to clinical practice. A prospective study might better explore these links.

Conclusion

The overrepresentation of children may result from two factors: their greater psychological vulnerability, but also greater vigilance about the suffering of children. The high level of patient attendance, adherence, and engagement in treatment suggest that TPT is an effective method for addressing the complex symptoms experienced by migrant families. This hypothesis will be clinically explored with an upcoming RCT protocol [60].

References

Buhmann CB, Carlsson J, Mortensen EL. Satisfaction of trauma-affected refugees treated with antidepressants and Cognitive Behavioural Therapy. Torture J. 2018;28: 118–129. doi:10.7146/torture.v28i2.106879

Lachal J, Moro MR. Assessment of Transcultural Psychotherapy in Child Major Depressive Disorder (Clinicaltrials.gov Identifier NCT04206969). Paris, France; 2019 Dec. Available: https://clinicaltrials.gov/ct2/show/NCT04206969

**Reviewer #3**

Thank you for the opportunity to review the manuscript entitled “An epidemiological description of 529 families treated by the French method of Transcultural Psychotherapy: A decade of experience”.

General Comments. The authors have carried out a retrospective study of 529 patients referred for the Transcultural Psychotherapy in Paris according to the French method. They classified the patients in three categories (no treatment, initiated treatment and effective treatment) and analyzed the differences between the initiated treatment and effective treatment group as well as the intercultural differences concerning clinical factors like reasons for attendance, main psychiatric symptoms and socio-demographic variables.

The study concerns an important issue in the field of mental health of migrants. The manuscript is well written, the methods and statistical analyses are appropriate. The results seem consistent and plausible. The discussion is clear and coherent, the conclusions are adequately drawn from the demonstrated data.

We would like to thank the reviewer for these overall comments.

Q1. I have only some minor points that should be considered in the revision:

Title:
As some of the patients had been classified to the group “no treatment” because they rejected the TPT for different reasons, the following statement in the title is not correct: “529 families treated by the French method of Transcultural Psychotherapy” -> please modify.

R1. We have modified the title, as suggested.

Modifications in the text:

Title: An epidemiological description of 529 families ~~treated by~~ the referred for French ~~method of Transcultural~~ Psychotherapy: A decade of experience

Q2. Abstract:

- Please add further aims: examination of intercultural differences and associations with socio-demographic and clinical variables.

- Please delete the word “descriptive” in the first sentence of the method because it can be misunderstood due to the fact that the presented study is not only a descriptive study because it reports not only descriptive statistics.

R2. We have made the modifications recommended.

Modifications in the text:

Abstract

This study aims to describe the patients referred for TPT in Paris and its suburbs over the past decade, to examine intercultural differences and associations with social, demographic, and clinical variables, and to assess TPT in terms of patient adhesion, attendance, and duration of care.

Method: Retrospective descriptive study of 529 patients referred for TPT care, classified in three categories – no treatment, initiated treatment, effectiveengaged and continuing treatment.

Q3. Introduction:

- p. 2: The authors state in the third sentence that “some authors characterize the Western mental health care system´s response to the needs of ethnically diverse populations as an overall failure”. But there are also other authors who recommend culture-sensitive (therapy) programs and services aiming for the optimization of mental health care for immigrants, e.g. in Canada or in Germany or other Western countries. Please add this information to the introduction with an example for such a culture-sensitive approach in the Western mental health care system.

R3. We added this point to the introduction

Modifications in the text:

Introduction

While some authors characterize the Western mental health care system's response to the needs of ethnically diverse populations as an overall failure [8], others recommend culture-sensitive therapy programs and services aiming for the optimization of mental health care for immigrants [9].

References

Kirmayer LJ. Cultural competence and evidence-based practice in mental health: Epistemic communities and the politics of pluralism. Soc Sci Med. 2012;75: 249–256. doi:10.1016/j.socscimed.2012.03.018

Q4. - p. 4: please report how large is the proportion of immigrants in the population in France and from which countries come the three largest immigrant collectives.

R4. We have provided some details about immigration to France.

Modifications in the text:

Patients come from diverse cultural backgrounds representative of the usual composition of immigration to France – 9.1% of the French population in 2014, with 31.5% from the European Union (EU), 30% from North Africa, 15% from sub-Saharan Africa, 15% from Asia, and 4% from European countries not members of the European Union [25].

Q5. Results:

- Table 1, last line: please place 228 to the right – to the column below N.

- Table 2: please change N to n in the columns Initiated follow-up group and Effective follow-up group.

- Table 2: please add the information regarding mean age (SD and range) of the children and adults and analyze gender-specific differences.

- Table 2: please provide an example for the categories Other healthcare professional and Other.

- Table 2: please add the data concerning the main psychiatric symptoms for children and for adults.

- Table 3: please provide for the variables generation, children and adults gender the information which is the reference category.

- Table 3: please report n for the different immigrant collectives.

- Table 3: the last column (Caribbean) is not visible -> please consider to change the format of this Table to horizontal format.

- Table 4: in the column Attendance please move mean to the line below.

- Table 4: please add the unit (months) to the column Treatment duration.

- S Table: please report the mean age (SD and range) of the children and adults for the different immigrant collectives.

R5. We have modified the tables in accordance with reviewer #3's recommendations and added stated the age- and gender-specific differences in the text.

Modifications in the text:

Please refer to Tables 1, 2, 3 and 4

Results

Women were referred more often than men (four women for every man) and the mean age of adults was 36.3 years (SD=12), with no significant difference between men (41.2, SD=12.8) and women (35.1, SD=11.6). ~~Among adults, the sex ratio was 25 (1 man to four women) and the mean age 36.3 years (12).~~

Boys and girls were referred at an equal rate (1:1), but the boys referred were significantly younger (12.5, SD=5.37) than the girls (14.5, SD=4.8) (p<0.01). ~~with a sex ratio around 100 (1:1).~~

Q6. - p. 9: “the sex ratio was 25”; … “around 100” – it is not clear what you mean, please reformulate.

- p. 9: “the mean age 36.3 years (12).” – what do you mean with (12) – SD? Please specify. (SD=12) (equally on page 13 – “82.3% (16)” –> (SD=16) ).

- p. 9: please consider to restructure the results section because now there is a frequent shift from Table 2 to Table 3 and vice versa.

R6. We have modified the two minor points and restructured the results section as requested.

Modifications in the text:

P9

Women were referred more often than men (four women for every man) and the mean age of adults was 36.3 years (SD=12), with no significant difference between men (41.2, SD=12.8) and women (35.1, SD=11.6). ~~Among adults, the sex ratio was 25 (1 man to four women) and the mean age 36.3 years (12).~~ First-generation migrants – the person who travelled – accounted for 93% of the adults.

Nearly half the children (48.5%) were the oldest child in their family, while second children accounted for 20.7% of the population younger than 18 years, third children 15.7%, and those ranked fourth to ninth in the family 15.1%. Mean age was 13.5 years old (SD=6.9). Boys and girls were referred at an equal rate (1:1), but the boys referred were significantly younger (12.5, SD=5.37) than the girls (14.5, SD=4.8) (*p*<0.01). ~~with a sex ratio around 100 (1:1).~~

Cultural area (Table 3)

We tested associations between cultural area of origin and socio-demographic and clinical variables.

We found no statistically significant associations between cultural area of origin and either age – i.e. adult vs children –, or gender among children or adults.

Multivariable regression models between cultural area of origin and cultural problems showed that Caribbean (OR=7.8) origins were associated with a higher risk of traditional theory problems, compared with patients from MENA (OR=0.5), Asia (OR=0.2), and Europe (OR=0.1). In contrast, Asian or European origins were associated with higher odds ratios for cultural misunderstandings (respectively OR=3.2 and OR=3), whereas MENA origins were associated with a higher odds ratio of traditional care problems (OR=4.7).

The distribution of psychiatric symptoms did not differ between cultural areas of origin, with the logistic regressions showing no statistically significant associations.

Finally, logistic regressions showed a statistically significant association between Asian origin and an interpreter's presence at the first session (OR=10.4).

P14

Attendance was high, with patients attending an average of 80% (SD=19.6) of their sessions; the rate was higher for those engaged in ~~effective~~ treatment, at 82.3% (SD=16). Attendance was not significantly associated with any of the study variables.

Q7. Discussion:

- p. 14: please provide the statement that living in two cultures can also mean benefits for the children (e.g. bi-cultural identity).

- p. 14: please provide also other reasons for the sex-ration differences among adults concerning the utilization of mental health services (e.g. women show a greater openness towards psychotherapy).

- p. 15: please provide an example for traditional theories.

- p. 17: please add the point to the limitations section that because of small sizes of two sub-samples (Europe and Caribbean) inter-ethnic differences probably could not be detected due to insufficient statistical power.

R6. We have completed the discussion with the points raised by all reviewers, and we have expanded the description of our limitation in accordance with the comments of all three reviewers.

Modifications in the text:

Discussion

Children and adolescents account for most of the patients referred. The experience of migration – encompassing the travel as well as the entire process of settlement and acculturation – stresses them more strongly than their parents and is correlated with more difficult psychological adjustment for them [31], despite the potential benefits to children of living in two cultures (e.g. a bicultural identity). Several vulnerability factors have been uncovered and differ between first- and second-generation migrant children.

…

These points may explain both the higher TPT referral rates for minors and the sex-ratio differential among adults. The latter result may also be explained by the more general underutilization of mental health services by men – native born or immigrant, in France and in many other countries.

…

The distribution of cultural problems may reflect the specificities of each cultural area: the proximity between French and other European cultures may explain the higher level of family conflicts and the lower level of traditional theories. Inversely, traditional theories around illness in sub-Saharan Africa and the Caribbean differ quite markedly from those in the West and rely most often on external causality – for example, mental suffering may be explained by possession or witchcraft. This point may explain the high proportion of issues involving traditional theories in these two subgroups. This result is in line with the literature: for example, Lanzara et al. found conflicting results about differences in prevalence of somatization between cultural groups [46].

…

Because of the small size of two subsamples (Europe and Caribbean) and thus insufficient statistical power, interethnic differences probably could not be detected.

Q8. References: Please translate French titles.

A8. We have now translated the French titles, as requested.

I hope my comments are helpful to the authors and to the editorial team.

We thank the reviewers for all points raised. We think that the revisions have enabled to improve the quality of the manuscript quite considerably.
