# Supplementary material for: Epidemiological description of 529 families referred for French transcultural psychotherapy: A decade of experience
Source: PLoS One. 2020 Aug 4;15(8):e0236990. doi: 10.1371/journal.pone.0236990 (PMC7402487; doi:10.1371/journal.pone.0236990)
Supplement: Attachment — Submitted filename: answer20200715.docx [file pone.0236990.s003.docx]

**Response to Reviewers**

An epidemiological description of 529 families treated by the French method of Transcultural Psychotherapy: A decade of experience

**Reviewer #3**

***Thank you very much for the revised version of the manuscript. Almost all my suggested improvements have been adequately implemented. There are three minor points that should be modified:***

***1. In the modified title "the" should be deleted.***

***2. Abstract: Please delete the word “descriptive” in the first sentence of the method because it can be misunderstood due to the fact that the presented study is not only a descriptive study because it reports not only descriptive statistics.***

***3. Please report in the text not only the ORs but also the CIs.***

We deleted “An” in the title and reports CI in the text. We checked but ‘descriptive’ was already deleted in the final abstract, maybe we made a copy/past mistake during the submission process.

**Reviewer #4**

***The authors provide a retrospective analysis of 529 families referred to French Transcultural Psychotherapy. The manuscript has been extensively revised based on previous reviewer comments and has benefitted from the revision. I only have a few minor additional points:***

***1) There are several grammar mistakes in the manuscript and in particular in the abstract that should be corrected.***

Sorry for that, the entire text was reread by a native speaker.

***2) Some information is scattered through the methods section and the results section a bit. In particular, the information on the time period of included referrals and the time period of data collection is distrubuted in an unfortunate way. I would suggest to summarize the time span of observation as well as the retrospective data collection in one paragraph once and not distributed over different sections.***

We moved the time period of data collection near the time span of observation.

***3) To me it is not clear why the rejected referral group is included in such a purely descriptive way. Wouldn´t a statistical comparison between rejected and treated referrals be a bit more interesting?***

Patients are referred to PTC from other institution, and we do not have access to medical files. The decision of treatment is made after a meeting-call with the therapist who refer the patient. Reason for refusal is the only data available for this group. We agree that comparison between the two group would be interesting, so we add this information in the limits of the study.

Modified text

No descriptive data were available for the rejected referral group, making comparison with the other groups impossible.
